# Case Report: A Novel Missense Mutation c.517G>C in the *UMPS* Gene Associated With Mild Orotic Aciduria

**DOI:** 10.3389/fneur.2022.819116

**Published:** 2022-03-09

**Authors:** Rui Ma, Jing Ye, Jiaqi Han, Lehong Gao, Chaodong Wang, Yuping Wang

**Affiliations:** ^1^Department of Neurology, Xuanwu Hosptial, Captial Medical University, Beijing, China; ^2^Beijing Key Laboratory of Neuromodulation, Beijing, China; ^3^National Clinical Research Center for Geriatric Diseases, Beijing, China

**Keywords:** case report, orotic aciduria, *UMPS*, epilepsy, intellectual disability

## Abstract

**Background:**

Hereditary orotic aciduria (HOA) is a rare genetic disorder of pyrimidine metabolism caused by variations in the uridine monophosphate synthetase (*UMPS*) gene and inheritance are autosomal recessive. Heterozygous *UMPS* mutations can also lead to orotic aciduria without clinical consequence.

**Methods:**

We conducted molecular genetic analyses on proband using whole-exome sequencing (WES) and on 12 family members using Sanger sequencing for *UMPS* mutation. We analyzed the urine metabolites of family members carrying *UMPS* heterozygous variants with standard gas chromatography-mass spectrometry (GC-MS).

**Results:**

We identified a novel *UMPS* mutation (c.517G>C) in a Chinese-origin of orotic aciduria pedigree. The proband presented with epilepsy and intellectual disability (ID). Other mutation carriers in our pedigree presented with mild orotic aciduria without relevant medical complaints except for the proband.

**Conclusion:**

Our study further expanded the genotype of orotic aciduria and highlighted the probability of misdiagnosis in clinical practice.

## Introduction

Hereditary orotic aciduria (HOA) (OMIM #258900) is a rare genetic disorder of pyrimidine metabolism characterized by early onset of megaloblastic anemia, global developmental delay, and failure to thrive, which is associated with massive urinary overexcretion of orotic acid (sometimes with orotic acid crystalluria) (1). Patients without megaloblastic anemia, but with additional manifestations such as epilepsy, have also been reported (2). The HOA is caused by variations in the uridine monophosphate synthetase (*UMPS*) gene with autosomal recessive inheritance. Heterozygous *UMPS* mutations can also lead to mild and isolated orotic aciduria without clinical consequence (1). Here, we discuss a mild orotic aciduria (OA) pedigree with a novel heterozygous *UMPS* mutation.

## Materials and Methods

### Editorial Policies and Ethical Considerations

This study was approved by the Xuanwu Hospital Capital Medical University, and all participants provided written informed consent.

### Patients and Family Members

**Figure 1:**
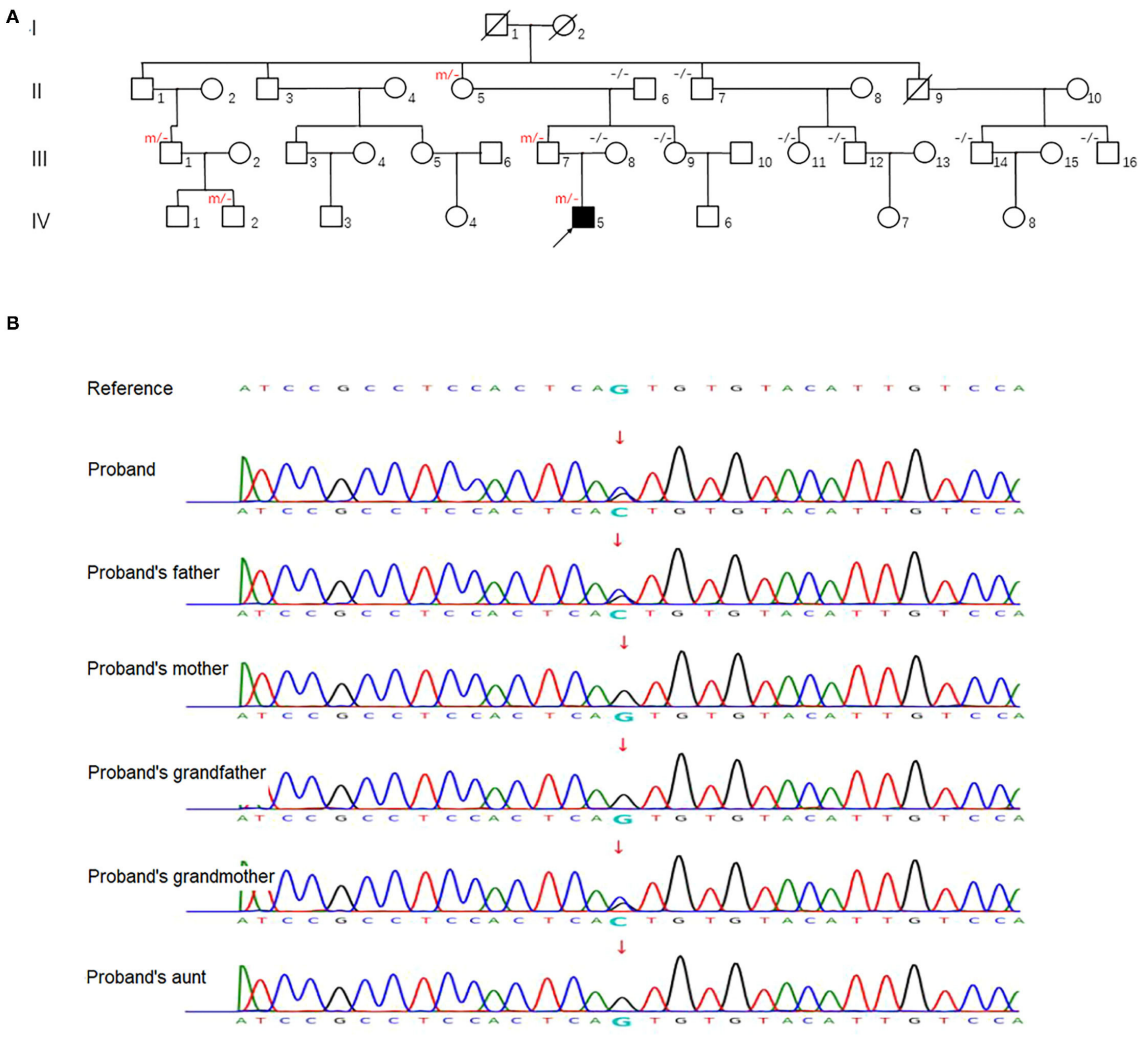
**(A)** Family pedigree. An arrow denotes the proband. “m/–” and “–/–” in the upper left indicates ascertained *UMPS* heterozygous mutation carrier and wildtype subject, respectively. **(B)** Sequence analysis of human *UMPS* gene in the proband and his family members. Representative sequence electropherograms are shown.

We investigated a large family with 36 members spanning four generations, originating from Beijing province, China (Figure 1A). Five family members, including the proband (IV-5), carried a heterozygous mutation in the *UMPS* gene. Family members without an *UMPS* mutation were included as controls.

### Genetic Tests

Genomic DNA was extracted from peripheral blood samples of the enrolled patient and their available family members using standard procedures. Variants were detected by next-generation sequencing using the NovaSeq600 platform by Berry Genomics (Beijing, China). Sanger sequencing with specific primers (5′-TTGAAGGTCACTGATGCCATAGT-3′ as the forward primer and 5′-GATTCGCTGCCACAAAGACA-3′ as the reverse primer) was performed to confirm the selected variant in the proband and other family members.

### Metabolic Screening Tests

Urine metabolites were analyzed by standard gas chromatography-mass spectrometry (GC-MS) as previously described (3).

## Results

### Case Report

The patient is a 16-year-old Chinese man who presented with epilepsy and intellectual disability. Pregnancy and delivery were uneventful. His parents are not consanguineous, and they had no relevant medical complaints. The first seizure, at the age of eight, was a generalized tonic-clonic seizure (GTCS) after he fell asleep. The second attack came 5 years later, presenting with limb weakness, numbness, head involuntary movement, and impaired awareness after a fever to a maximum of 38.4°C. Paroxysmal unilateral limb numbness with face numbness, dizziness, double vision, and slurred speech occurred approximately twice a year after the age of 14. Wechsler Intelligence Scale for Children (WISC) was tested at the age of eight, indicating mild intelligent disability (intelligence quotient 68). Several electroencephalograms (EEG)s showed paroxysmal generalized spike and slow-wave, prominent in the posterior head. No abnormality was observed in the head MRI. The patient did not have blood count abnormalities, relevant hyperammonemia, or altered plasma amino acid profile except a reduced level of Vitamin B12 (161 pg/ml, reference range 180-914 pg/ml). The patient's parents refused to take antiepileptic drugs and uridine triacetate except for mecobalamin. None of these episodes occurred at a one-year follow-up.

### Genetic and Metabolic Screening Analysis

**Figure 2:**
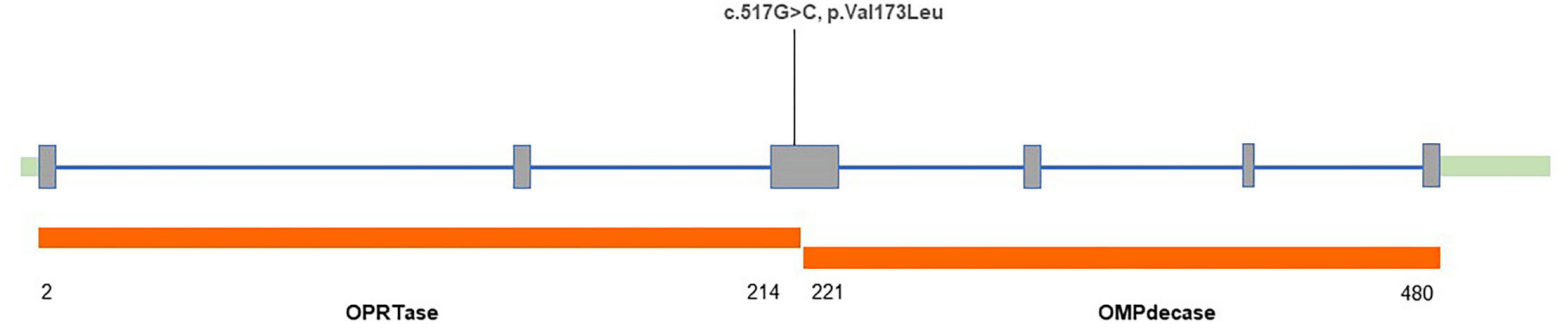
Structure of the human uridine monophosphate synthetase (UMPS*)* gene with variant found in our patient.

After obtaining informed consent, blood samples were collected and processed for genomic DNA isolation. Whole-exome sequencing (WES) was performed using a trio-based approach (patient, mother, and father). The analysis demonstrated a previously unreported heterozygous mutation inherited from his father in the *UMPS* gene (c.517G>C, p.Val173Leu, reference sequence: NM_000373.3), which have not yet been reported in the literature. These sequence data have been submitted to the GenBank databases under accession number OK605588. Parents were clinically asymptomatic. Subsequent Sanger sequencing of all available paternal family members found that III-7, II-5, III-1, and IV-2 also carried *UMPS* variant (c.517G>C, p.V173L) with no neurological symptoms (Figures 1A,B). The mutation located in the orotate phosphoribosyltransferase domain was not found in The Genome Aggregation (GnomAD), The Exome Aggregation (ExAC), and 1,000 Genomes (1,000G) databases. Prediction tools [Sorting Intolerant from Tolerant (SIFT) and Polymorphism Phenotyping v2 (PolyPhen-2)] were suggested for c.517G>C (p.V173L), a minor effect on enzymatic activity. Mutation Taster considered the mutation disease-causing. Based on the American College of Medical Genetics and Genomics (ACMG) standards and guidelines, c.517G>C of the *UMPS* gene was predicted to be likely pathogenic (PS3+PM1+PM2). The location of the mutation in the schematic diagram shown in Figure 2.

Further findings of a significant urinary excretion of orotate in our patient at the age of 16 (elevated 16.739 times of reference value) supported the diagnosis of orotic aciduria. Values of urinary excretion of orotate of family members carrying *UMPS* heterozygous variant are shown in Table 1. Except for III-1, our patient's paternal uncle, the ratios of analysis value to reference value of other family members with *UMPS* variant are more significant than one, indicating the diagnosis of orotic aciduria. According to the results of metabolic screening analysis, four family members (III-7, II-5, IV-2, and the proband) in this family can be diagnosed as orotic aciduria, although with no neurological symptoms.

**Table 1:** Value of urinary excretion of orotate of family members carrying uridine monophosphate synthetase (UMPS*)* heterozygous variant.

**Number**	**Clinical signs and symptoms**	**Age of test/year**	**Analysis value**	**Reference value**	**Ratio**
V-5	Epilepsy and intellectual disability	13	0.0479	0.023	2.083
		16	0.3850		16.739
III-7	NRMC	43	0.0556		2.417
II-5	NRMC	70	0.0366		1.591
III-1	NRMC	45	0.011		0.483
IV-2	NRMC	12	0.027		1.174

*NRMC no relevant medical complaints*.

## Discussion

The HOA results from deficiencies in uridine monophosphate synthetase (UMPS), which consists of two domains of the enzyme: orotate phosphoribosyl pyrophosphate transferase (OPRTase, EC 2.4.2.10) and orotidine monophosphate decarboxylase (OMPdecase, EC 4.1.1.23). According to the values of urinary orotic acid and orotidine excretion, the type of UMPS deficiency can be presented *in vivo* (4): Type I is caused by the loss of both enzyme activities of the UMP enzyme; Type II is thought to be due to the specific inactivation of OMPdecase; Type III, orotic aciduria without megaloblastic anemia (OAWA), is expected to be also secondary to the inactivation of OMPdecase. Urinary orotidine levels were not available in our pedigree, so the type of OA cannot be further classified.

To the best of our knowledge, this *UMPS* nonsynonymous mutation (c.517G>C) had never been identified in the single-nucleotide polymorphism database (dbSNP), Leiden Open Variation Database (LOVD) (v3.1), and ClinVar (https://www.ncbi.nlm.nih.gov/clinvar/) databases. There are previous reports of patients with seizure-associated HOA (2, 5), thus, proposing a link between UMPS deficiency and epilepsy. Intellectual disability occurs in patients with OA with homozygous and heterozygous *UPMS* mutations (1). Moreover, our OA proband presented with epilepsy, intellectual disability, and vitamin B12 deficiency, which is partly consistent with previous reports. Wortmann et al. (1) reported 11 index cases and 18 healthy heterozygous mutation carriers, revealing high phenotypic heterogeneity of *UMPS* mutations. They thought that presentation of developmental delay or intellectual disability in their patient population might be due to ascertainment bias and *in utero* exposure to elevated levels of orotic acid and/or decreased pools of pyrimidines. However, our proband had a paternally derived mutation and did not support the utero exposure hypothesis. The other four *UPMS* mutation carriers in our family all presented with no neurologic complaints. Three of them had mild orotic aciduria, which also confirms the high phenotypic heterogeneity of *UMPS*. Significant urinary excretion of orotate was detected in those members who carried this mutation by urine metabolic screening in our research. Orotate is a substrate of orotate phosphoribosyl pyrophosphate transferase and orotidine monophosphate decarboxylase, which are encoded by the *UMPS* gene. Therefore, the excessive accumulation of orotate can reflect the impaired function of the *UMPS* gene and augment the value of this case report (6).

In 2015, the U.S. Food and Drug Administration approved a treatment for HOA called uridine triacetate (Xuriden), which is a pyrimidine analog indicated for uridine replacement therapy (7). Patients who took the drug responded well (5). Although our proband only took mecobalamin, he was seizure-free for one year. We will continue to pay attention to the patient's condition and adjust the treatment plan according to the condition at any time.

## Conclusion

Although extremely rare, hereditary orotic aciduria should be suspected in any child with neurological disorders, such as epilepsy and developmental delay, and with or without anemia. Our study characterizes a highly heterogeneous *UMPS* pedigree, expanding the mutational spectrum as we report one novel variant that is identified in the *UMPS* gene. Early diagnosis and treatment are essential for disease course.

## Data Availability Statement

The datasets presented in this article are not readily available due to ethical and privacy restrictions. Requests to access the datasets should be directed to the corresponding author.

## Ethics Statement

The studies involving human participants were reviewed and approved by the Ethical Committee of Xuanwu Hospital Capital Medical University. Written informed consent to participate in this study was provided by the participants' legal guardian/next of kin. Written informed consent was obtained from the individual(s), and minor(s)' legal guardian/next of kin, for the publication of any potentially identifiable images or data included in this article.

## Author Contributions

RM: writing—original draft, visualization. JY: resources. JH: investigation. LG: writing—reviewing and editing, validation. CW: data curation and conceptualization. YW: supervision, project administration, and funding acquisition. All authors contributed to the article and approved the submitted version.

## Funding

This study was supported by the National Key R&D Program (No. 2018YFC1314500, 2018YFC1314504) and the Clinical Cohort Study of Epilepsy Patient (2017YFC0907702).

## Conflict of Interest

The authors declare that the research was conducted in the absence of any commercial or financial relationships that could be construed as a potential conflict of interest.

## Publisher's Note

All claims expressed in this article are solely those of the authors and do not necessarily represent those of their affiliated organizations, or those of the publisher, the editors and the reviewers. Any product that may be evaluated in this article, or claim that may be made by its manufacturer, is not guaranteed or endorsed by the publisher.
